# Supplementary material for: Image-based phenotypic sorting of synthetic cells
Source: Sci Adv. 2026 Jun 3;12(23):eaed0860. doi: 10.1126/sciadv.aed0860 (PMC13232557; doi:10.1126/sciadv.aed0860)
Supplement: Supplementary file 1 — Figs. S1 to S10 Tables S1 to S14 DNA sequences Legend for data S1 [file sciadv.aed0860_sm.pdf]

Science Advances
AAAS

Supplementary Materials for

Image-based phenotypic sorting of synthetic cells

Marijn van den Brink *et al.*

Corresponding author: Christophe Danelon, danelon@insa-toulouse.fr

Sci. Adv. **12**, eaed0860 (2026)
DOI: 10.1126/sciadv.aed0860

The PDF file includes:

Figs. S1 to S10
Tables S1 to S14
DNA sequences
Legend for data S1

Other Supplementary Material for this manuscript includes the following:

Data S1

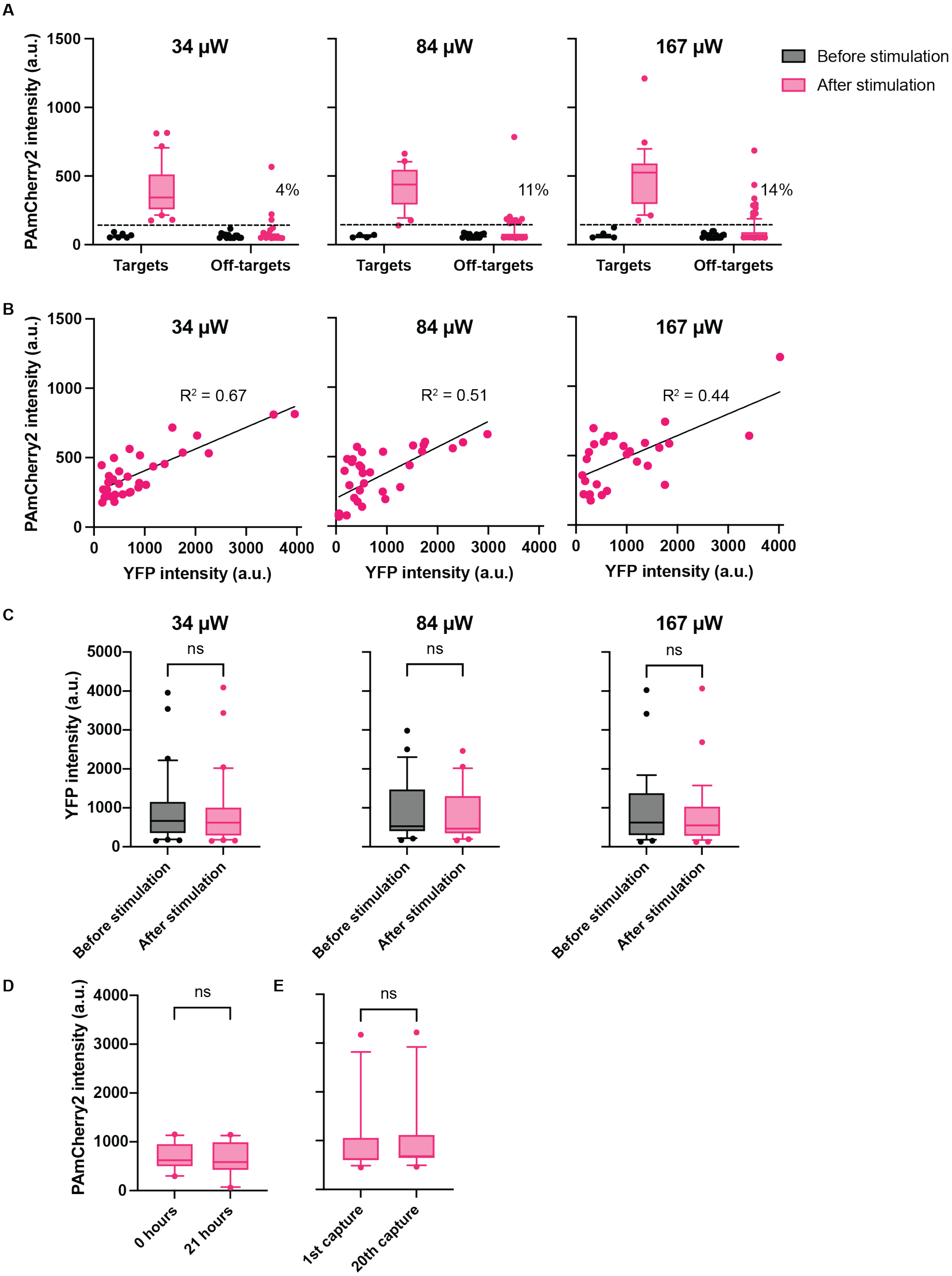

Fig. S1. PAmCherry2 and YFP fluorescence intensities before and after liposome stimulation. YFP was expressed in liposomes containing 5 nM *YFP* DNA template and 20 μM purified PAmCherry2. Liposomes were stimulated in the center of the FOV for 300 ms using different laser powers (34, 84 and 167 μW). The fluorescence intensity after stimulation was measured 10 s after stimulation. (**A**) PAmCherry2 fluorescence intensity in target and off-

target liposomes before and after stimulation. All target liposomes were successfully tagged for the three photoactivation conditions with no significant differences in the median intensity values (Mann-Whitney test, P = 0.38 for 34 μW vs 84 μW; P = 0.11 for 34 μW vs 167 μW). All liposomes within 15 μm of the target liposome were considered as off-targets. Per condition, 29-31 target liposomes and 78-94 off-target liposomes were measured. The percentages of off-target liposomes with increased PAmCherry2 intensity (above a certain threshold indicated with the dashed line) relative to all off-target liposomes are appended in the graphs. The data for the target liposomes of the 84 μW condition are also shown in Fig. 2A. (**B**) Positive correlation between the YFP intensity (before stimulation) and the PAmCherry2 intensity after stimulation (Pearson's r = 0.82 for 34 μW; r = 0.72 for 84 μW; r = 0.67 for 167 μW; P < 0.0001 for all). A linear regression curve is shown in the graphs with the corresponding R^2 value. Per condition, 29-31 target liposomes were measured. For the 84 μW condition, ten additional liposomes were measured with low YFP intensity (< 65 a.u.) and low PAmCherry2 intensity (< 95 a.u.) values. (**C**) YFP intensities before and after stimulation were not significantly different (unpaired t test, P = 0.88, t = 0.1540, degrees of freedom = 60 for 34 μW; P = 0.50, t = 0.6778, degrees of freedom = 56 for 84 μW; P = 0.52, t = 0.6528, degrees of freedom = 56 for 167 μW), thus no significant YFP bleaching occurred. Per condition, 29-31 target liposomes were measured. (**D**) PAmCherry2 fluorescence values are constant up to 21 hours in liposomes. Twelve YFP-expressing liposomes were activated (34 μW laser power, 300 ms) and tracked over time by imaging every hour. PAmCherry2 intensities at t0 and t21 h were not significantly different (unpaired t test, P = 0.71, t = 0.3782, degrees of freedom = 22). (**E**) Control experiment for panel d. PAmCherry2 was not bleached due to repeated imaging (20 captures within 30 min) (unpaired t test, P = 0.84, t = 0.2027, degrees of freedom = 22). Twelve target liposomes were measured.

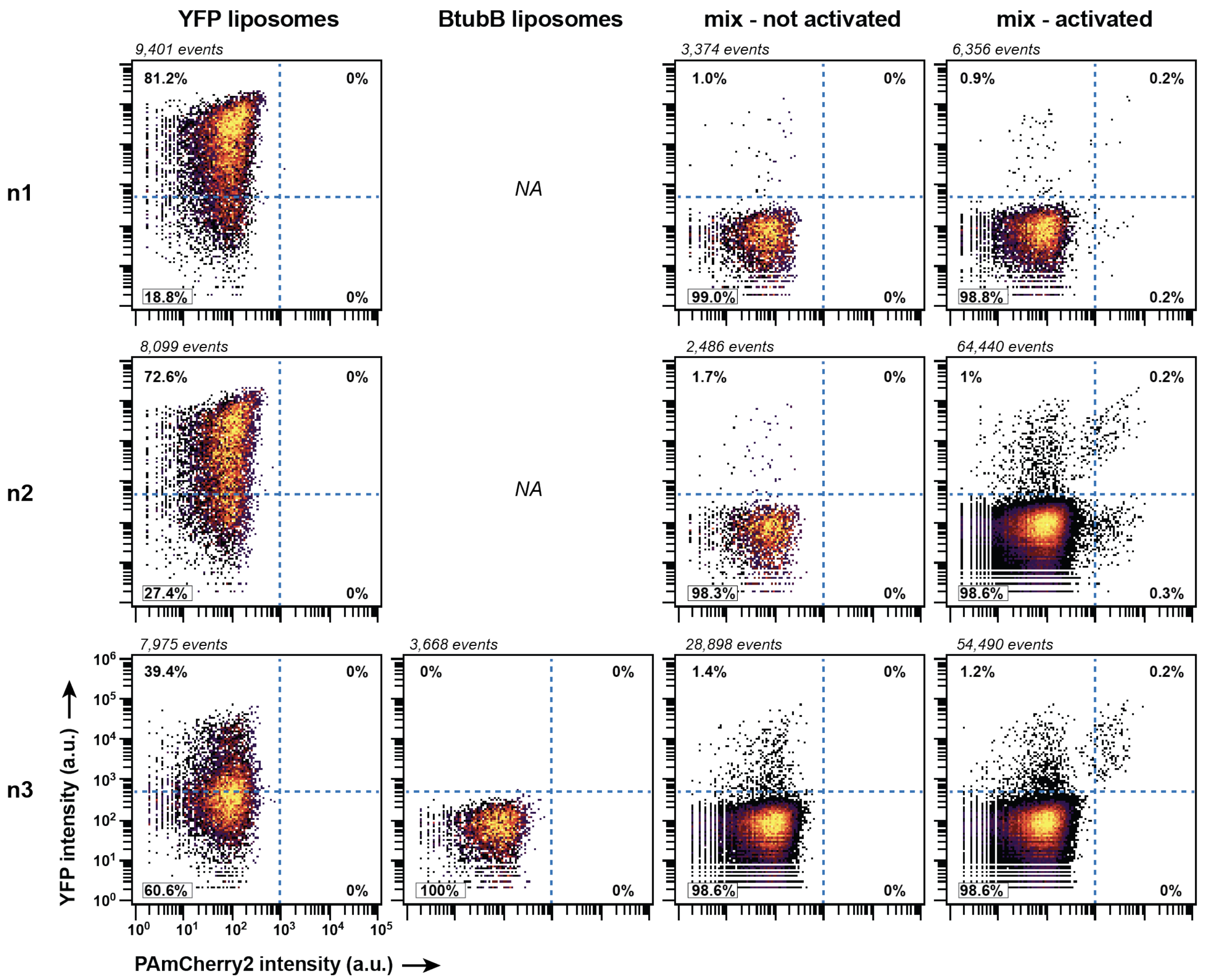

Fig. S2. Flow cytometry measurements of (a mix of) YFP- and BtubB-expressing liposomes, without or with activation of PAmcherry2. This figure supplements Fig. 2E with three biological replicates. The two right-most plots from replicate 3 (n3) are also shown in Fig. 2E. The number of events varies per plot as indicated above the graphs. The percentage of liposomes in each gate is also indicated. Experiment 3 (n3) was performed with laser power 34 μW and stimulation in the center of the FOV, while experiments 1 and 2 (n1-2) were performed with laser power 84 μW and stimulation was not restricted to the center of the FOV, likely explaining the lower stimulation precision for experiments 1-2.

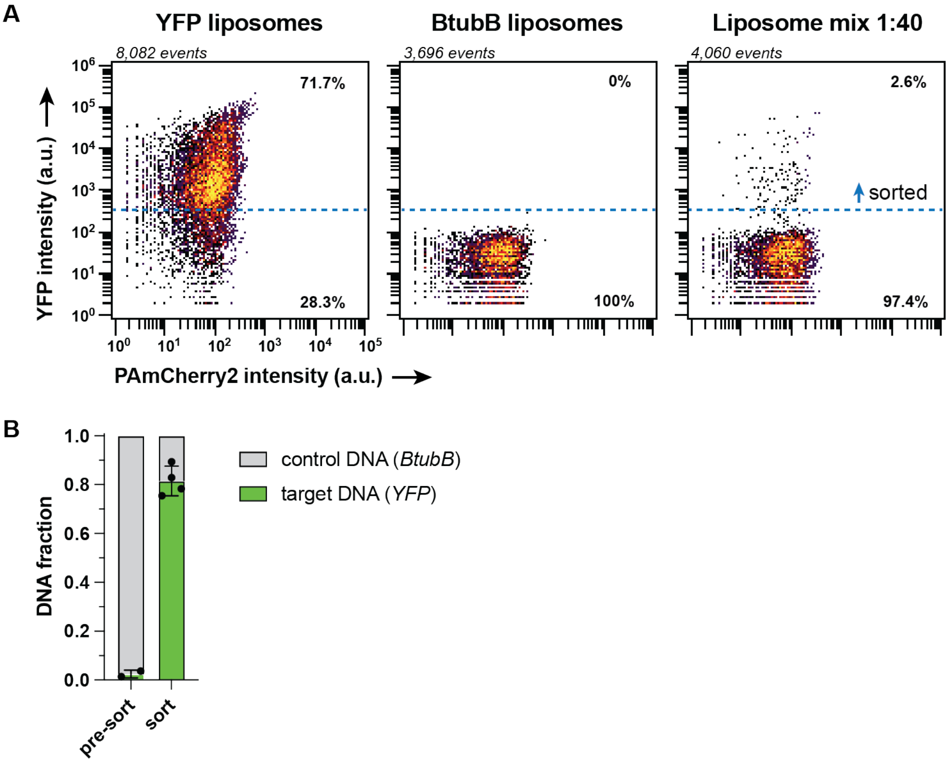

Fig. S3. FACS sorting of YFP-expressing liposomes from a mixed population of YFP- and BtubB-expressing liposomes based on YFP fluorescence intensity. (**A**) FACS plots showing YFP intensity vs PAmCherry2 intensity for one of two biological replicates. The second replicate corresponds to the left three plots of n3 in Fig. S2. Number of events are indicated above the plots. The percentage of liposomes in each gate is also indicated. (**B**) qPCR analysis of the sorted liposomes showing enrichment of YFP DNA upon sorting. Two biological replicates (mixed liposome samples) with two technical replicates (sorts) each.

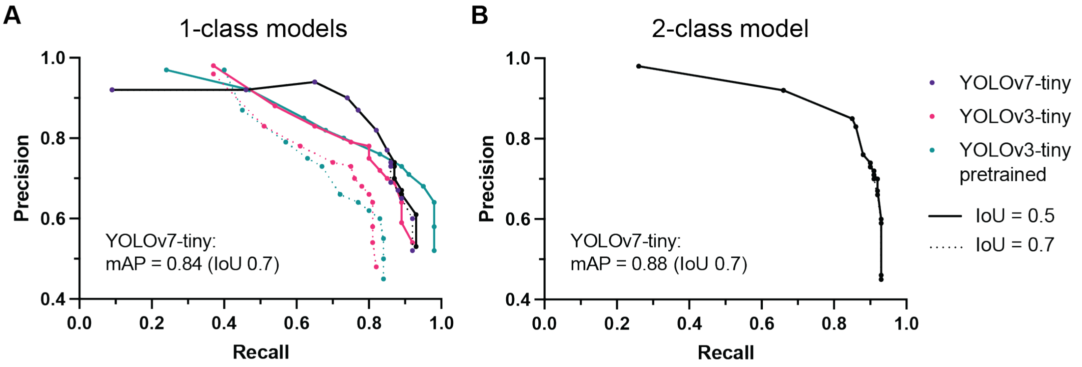

Fig. S4. Performance of trained YOLO-tiny models. (**A**) YOLO-tiny models trained on one class to detect liposomes with eGFP-MinC at the membrane. mAP is mean Average Precision, and IoU is Intersection over Union. For YOLOv7-tiny (trained from scratch), YOLOv3-tiny (trained from scratch) and YOLOv3-tiny (pretrained), the mAP is 0.85, 0.85 and 0.87 for IoU 0.5, and 0.84, 0.73 and 0.72 for IoU 0.7, respectively. The mAP remains very similar for YOLOv7-tiny when increasing the IoU from 0.5 to 0.7, while for YOLOv3-tiny the mAP decreases. Therefore, the YOLOv7-tiny model has better localization performance of the bounding boxes than the YOLOv3-tiny models. The trained YOLOv7-tiny model was used in liposome experiments at a confidence threshold of 0.8, with both a precision and recall of 82% (IoU 0.5 and 0.7). The YOLOv7-tiny data are also shown in Fig. 3C. (**B**) YOLOv7-tiny model trained on two classes to detect liposomes with eGFP-MinC at the membrane, and liposomes with eGFP-MinC in the lumen. mAP is 0.88 for both IoU 0.5 and 0.7. The model was used at a threshold of 0.9 (precision 83%, recall 86%).

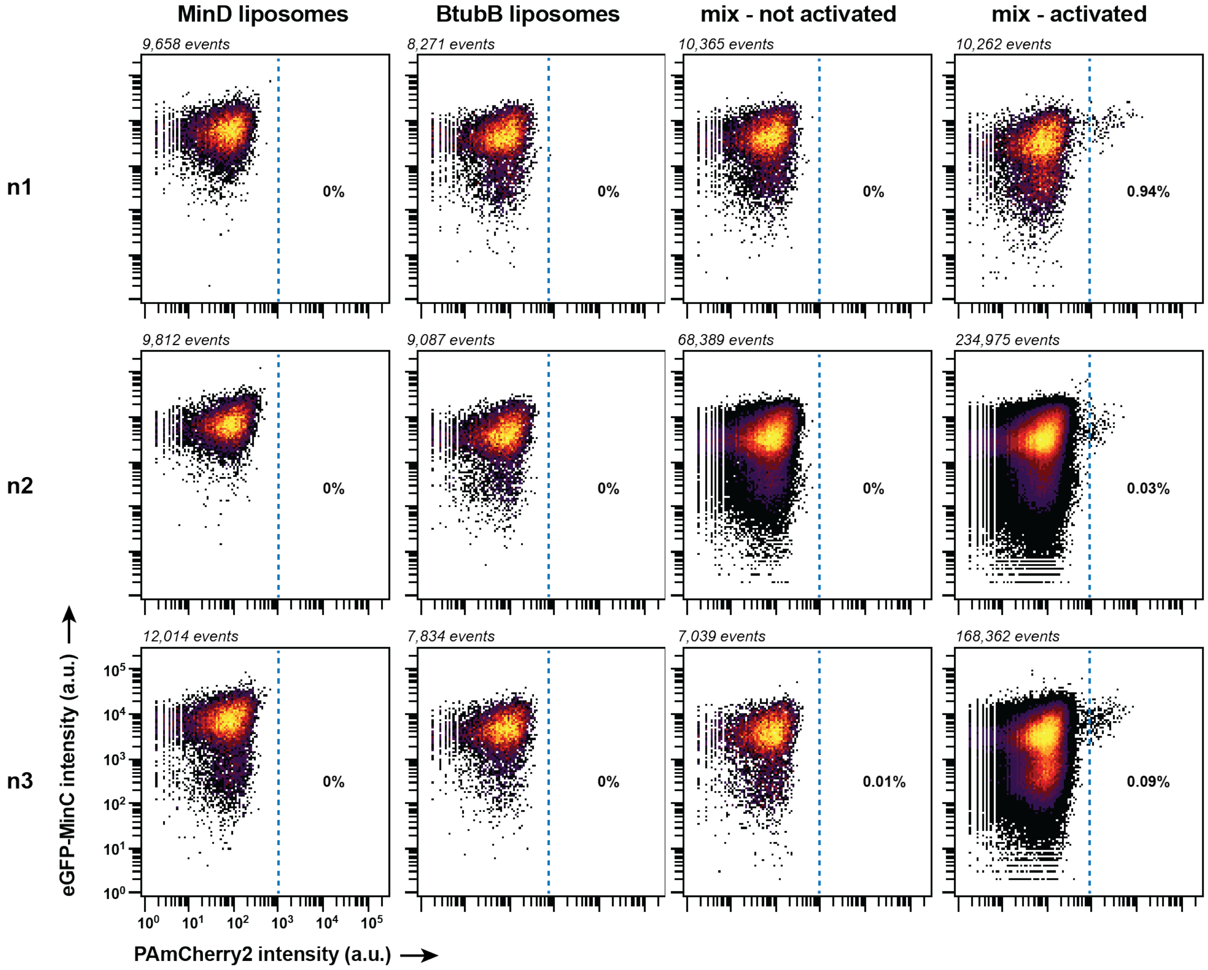

Fig. S5. Flow cytometry measurements of (a mix of) MinD- and BtubB-expressing liposomes, without or with activation of PAmcherry2. This figure supplements Fig. 3E with three biological replicates. The two right-most plots from replicate 1 (n1) are also shown in Fig. 3E. The number of events varies per plot as indicated above the graphs. The percentage of liposomes in each gate is also indicated.

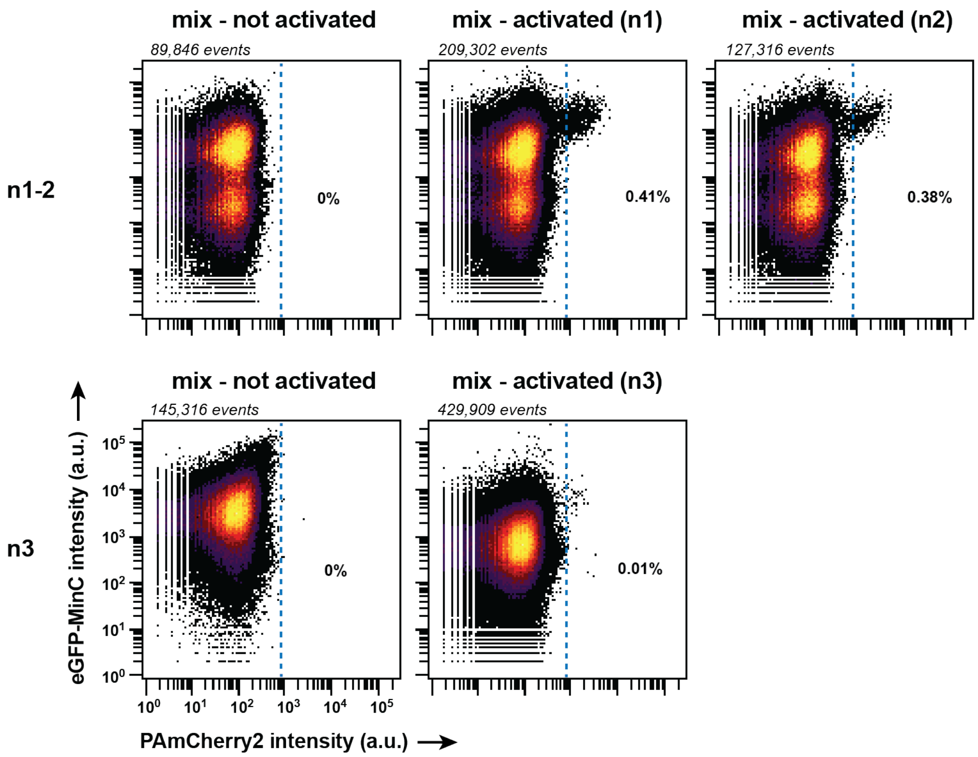

Fig. S6. Flow cytometry measurements of liposomes expressing a DNA library encoding MinD with mutations in the MTS, without or with activation of PAmcherry2. This figure supplements Fig. 5. Two biological replicates, for which the first replicate entailed two independent sorts (n1 and n2) and the second replicate entailed one sort (n3). The number of events varies per plot as indicated above the graphs. The percentage of liposomes in each gate is also indicated.

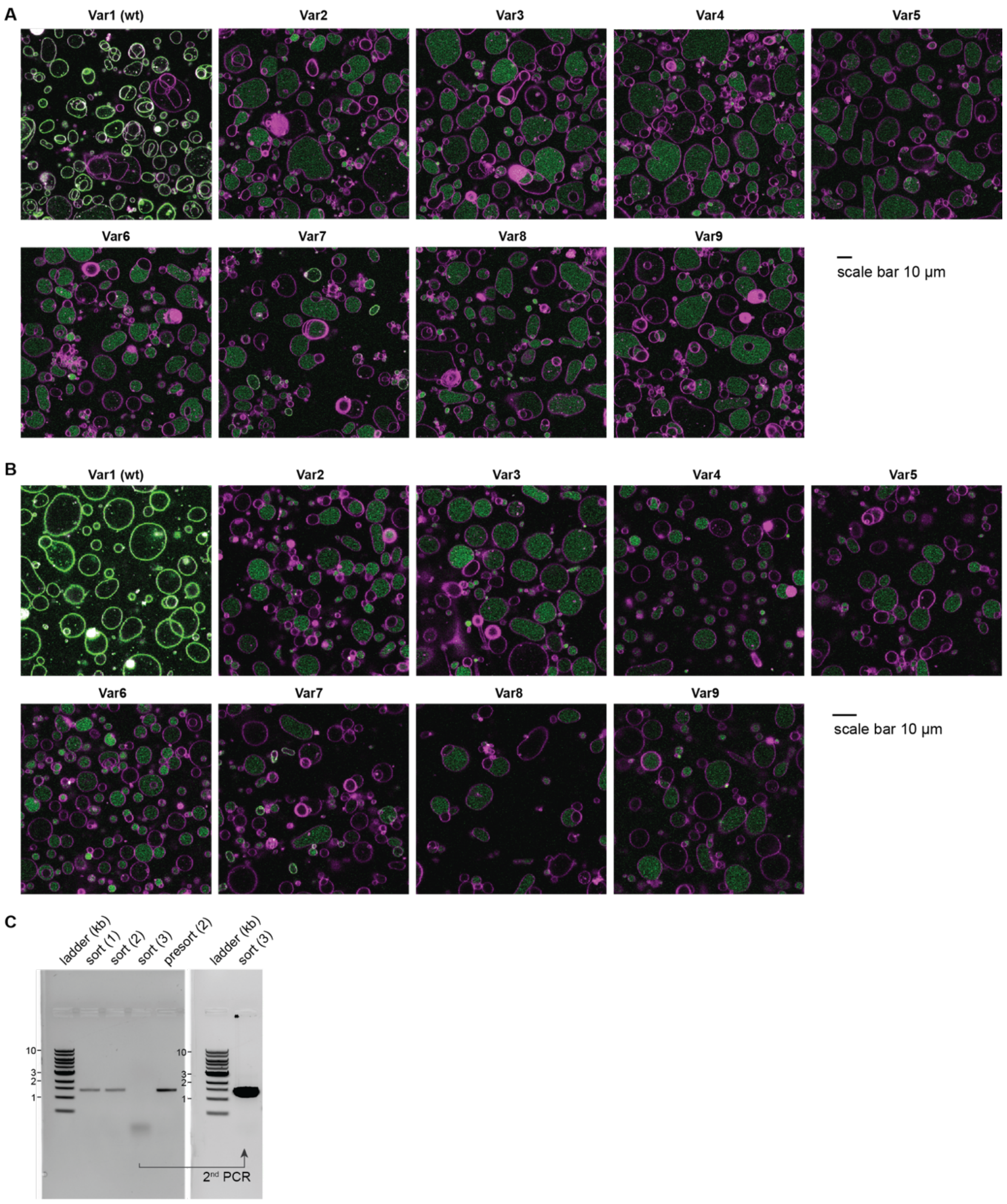

Fig. S7. Additional data for the library of MinD MTSs. (**A-B**) Fluorescence confocal microscopy images of liposomes expressing clonal DNA variants with mutations in the MinD MTS. Green, eGFP-MinC; magenta, Cy5-conjugated lipids. Scale bars: 10 μm. In replicate 1 (**A**), the liposomes were diluted 15-fold before imaging using PB buffer. In replicate 2 (**B**), the liposomes were diluted 15-fold using a 1:1 mixture of PURE*frex* 2.0 Solution I and MilliQ. The images for Var1 (wt) and Var 7 in replicate 1 (**A**) are also presented in Fig. 5E. (**C**) Gel electrophoresis with PCR-amplified *minD* (1,398 bp) recovered from sorted liposomes. For sort 3, the full-length DNA was recovered using two subsequent, identical PCRs, where the second PCR contained 1 μL of the first PCR as the source of DNA template instead of the sorted liposomes.

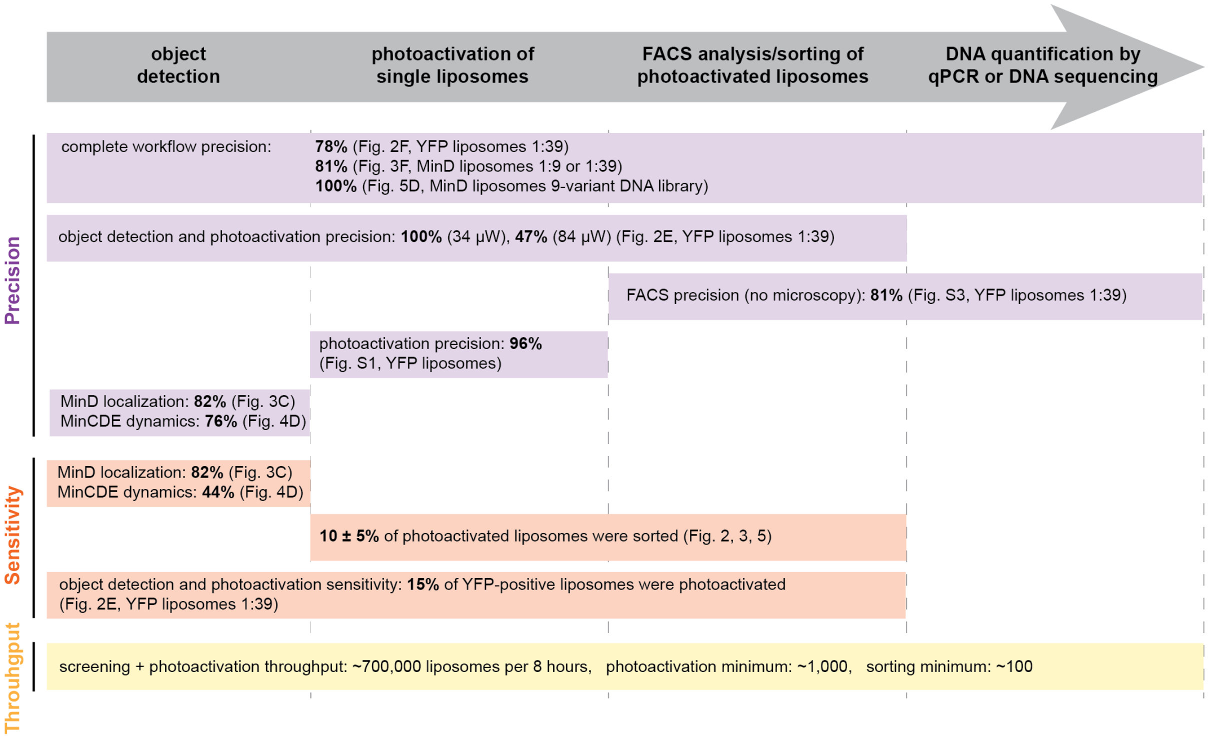

Fig. S8. Precision, sensitivity and throughput parameters of the method presented in this work. The precision, sensitivity and throughput were extensively quantified for the different stages in the workflow. A comparison of the metrics to those of other studies is provided as separate supplementary material: Supplementary Data 1.

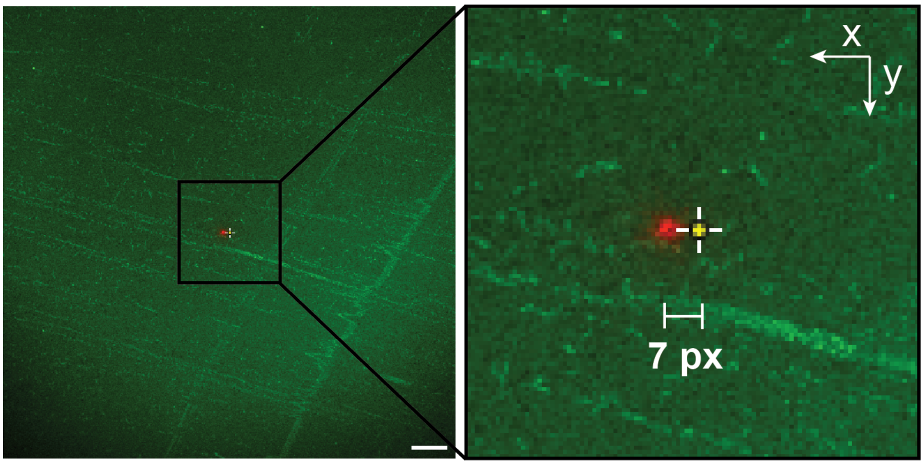

Fig. S9. Difference between the target and observed location of point-stimulation. mEos2 protein was imaged at the glass surface of a cover slip and point-stimulated in the center pixel of the field of view (405 nm, 300 ms, 5 µW laser power). The difference in xy location between the target stimulation point (yellow dot) and the observed green-to-red photoconversion and bleaching of mEos2 was quantified (here: 7 pixels in the x-direction, i.e., 1.75 µm). The differences in x and y locations were corrected for in the JOBS protocol (Nis-Elements software). Scale bar: 10 µm.

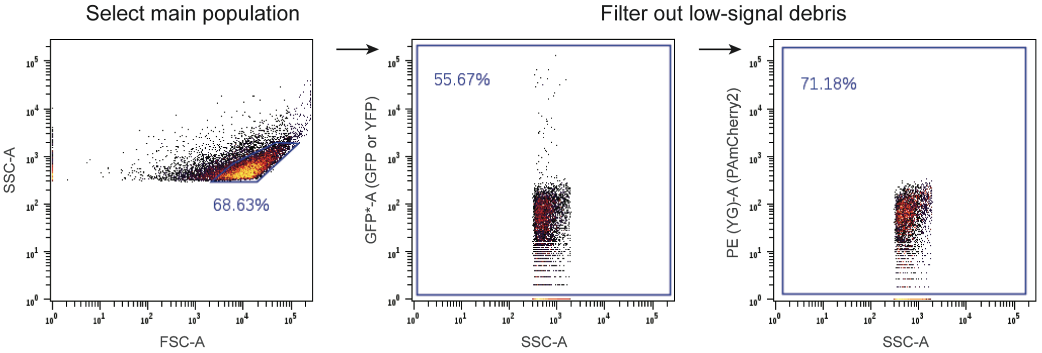

Fig. S10. Example of gating strategy applied to FACS data. While sorting liposomes, only the first step (gating based on forward and side scatter light) was applied. Post-measurement analysis involved all three filtering steps.

Table S1. Experimental details for photoselection and sorting of liposomes from *YFP-btubB* mock library. The number of stimulated liposomes was estimated by counting the stimulated liposomes for a fraction of all images.

Exp	Exp ID	Stim at center FOV	Laser power (μW)	Stim time (ms)	Correction xy point stimulation	Number of FOVs screened	Number of stimulated liposomes	FACS sorting threshold	Number of sorted liposomes	Initial ratio *YFP:btubB*
1	20230713	no	84	300	no	1,914	870	1,000	193	1:40
2	20230721	no	84	300	no	4,687	7,218	1,800	856	1:40
3	20240429	yes	34	300	yes, 7 px in x direction	3,440	1,668	910	154	1:50

Table S2. Percentages of gated liposomes (extended data for Fig. S2). Gating thresholds for PAmCherry2 signal and YFP were 988 a.u. and 542 a.u., respectively. ROI 1-4 are the top left, top right, bottom left and bottom right gates, respectively.

	YFP liposomes				BtubB liposomes				Mix - not activated				Mix - activated			
**Exp**	**ROI 1**	**ROI 2**	**ROI 3**	**ROI 4**	**ROI 1**	**ROI 2**	**ROI 3**	**ROI 4**	**ROI 1**	**ROI 2**	**ROI 3**	**ROI 4**	**ROI 1**	**ROI 2**	**ROI 3**	**ROI 4**
1	81.16%	0.01%	18.83%	0.00%	*NA*	*NA*	*NA*	*NA*	0.98%	0.00%	99.02%	0.00%	0.93%	0.17%	98.75%	0.15%
2	72.63%	0.00%	27.37%	0.00%	*NA*	*NA*	*NA*	*NA*	1.73%	0.00%	98.27%	0.00%	1.02%	0.18%	98.55%	0.25%
3	39.39%	0.00%	60.61%	0.00%	0.00%	0.00%	100.00%	0.00%	1.38%	00.0%	98.62	0.00%	1.21%	0.22%	98.57%	0.00%

Table S3. DNA concentrations in the sorted fractions of the *YFP-btubB* mock library as measured by qPCR.

	[*btubB*] (pM)		[*YFP*] (pM)		*YFP* fraction	
**Exp**	**Pre-sort**	**Post-sort**	**Pre-sort**	**Post-sort**	**Pre-sort**	**Post-sort**
1	0.030018	0.000287	0.000756	0.001045	0.0245	0.7846
2	0.030467	0.000754	0.001257	0.001159	0.0396	0.6060
3	0.101736	0.000003$	0.001403	0.000051	0.0136	0.83507* -0.9525$

* when taking the lowest calibration curve data point (0.00001 pM)

$ if calibration curve was forced to extend below the lowest calibration data point (0.00001 pM)

Table S4. Experimental details for sorting liposomes from *YFP-btubB* mock library based on the YFP signal (liposomes were not stimulated in microscope). Data consist of 2 biological replicates (mock library liposome mixes), each containing 2 technical sorting replicates.

Exp	Exp ID	FACS sorting threshold	Number of sorted liposomes	Initial ratio *YFP:btubB*
1	20230912	350	7,021	1:40
		350	5,006	
2	20240429	418	513	1:50
		418	1,338	

Table S5. Percentages of gated liposomes (extended data for Fig. S3). Gating thresholds for PAmCherry2 signal and YFP were 988 a.u. and 542 a.u., respectively. ROI 1-4 are the top left, top right, bottom left and bottom right gates, respectively.

	YFP liposomes				BtubB liposomes				Mix - not activated			
Exp	ROI 1	ROI 2	ROI 3	ROI 4	ROI 1	ROI 2	ROI 3	ROI 4	ROI 1	ROI 2	ROI 3	ROI 4
1	71.74%	0.00%	28.26%	0.00%	0.00%	0.00%	100.00%	0.00%	2.62%	0.00%	97.38%	0.00%
2	39.39%	0.00%	60.61%	0.00%	0.00%	0.00%	100.00%	0.00%	1.38%	00.0%	98.62	0.00%

Table S6. DNA concentrations in the sorted fractions of the YFP-BtubB mock library as measured by qPCR. Liposomes were not stimulated in microscope but sorted based on the YFP signal.

	[*btubB*] (pM)		[*YFP*] (pM)		*YFP* fraction	
Exp	Pre-sort	Post-sort	Pre-sort	Post-sort	Pre-sort	Post-sort
1	0.190905	0.002459	0.007178	0.008871	0.0362	0.7829
		0.001243		0.010397		0.8932
2	0.101736	0.000012	0.001403	0.000037	0.0136	0.7542
		0.000019		0.000090		0.8289

Table S7. Experimental details for photoselection and sorting of liposomes from *minD-btubB* mock library. The number of stimulated liposomes for experiment 1 was estimated by counting the stimulated liposomes for a fraction of all images. The exact numbers are presented for experiments 2 and 3.

Exp	Exp ID	Stim at center FOV	Laser power (μW)	Stim time (ms)	Correction xy point stimulation	Number of FOVs screened	Number of stimulated liposomes	FACS sorting threshold	Number of sorted liposomes	Initial ratio *minD:btubB*
1	20231206	yes	84	300	Yes, 7 px in x direction	2,880	8,640	1,000	905	1:9
2	20240503	yes	34	300	Yes, 7 px in x direction	5,160	1,797	700	171	1:40
3	20240508	yes	34	300	Yes, 7 px in x direction	6,880	3,893	700	189	1:40

Table S8. Percentages of gated liposomes (extended data for Fig. S5). Gate > 988 a.u (PAmCherry2 signal). Percentages based on dataset with most events.

Exp	MinD liposomes	BtubB liposomes	Mix - not activated	Mix - activated
1	0.00%	0.00%	0.00%	0.94%
2	0.00%	0.00%	0.00%	0.03%
3	0.00%	0.00%	0.01%	0.09%

Table S9. DNA concentrations in the sorted fractions of the *minD-btubB* mock library as measured by qPCR.

	[*btubB*] (pM)		[*minD*] (pM)		*minD* fraction	
**Exp**	**Pre-sort**	**Post-sort**	**Pre-sort**	**Post-sort**	**Pre-sort**	**Post-sort**
1	0.031877	0.000262	0.005609	0.000437	0.1496	0.6253
2	0.244735	0.000030	0.007848	0.000144	0.0311	0.8274
3	0.399657	0.000005	0.016422	0.000535	0.0395	0.9817*

* when taking the lowest calibration curve data point (0.00001 pM)

Table S10. Experimental details for photoselection of liposomes displaying Min oscillations.

Exp	Exp ID	Stim at center FOV	Laser power (μW)	Stim time (ms)	Correction xy point stimulation	Number of FOVs screened	Number of stimulated liposomes	Time of experiment (h)
1	20241009	yes	34	300	Yes, 11 px in x direction	170	89	3.3
2	20241113	yes	34	300	Yes, 11 px in x direction	401	190	7.8
3	20241203	yes	34	300	Yes, 11 px in x direction	337	146	7.9

Table S11. Experimental details for photoselection of liposomes from the MinD library with mutations in the MTS.

Exp	Exp ID	Stim at center FOV	Laser power (μW)	Stim time (ms)	Correction xy point stimulation	Number of FOVs screened	Number of stimulated liposomes	FACS sorting threshold	Number of sorted liposomes
1	20240522	yes	34	300	Yes, 7 px in x direction	1,720	6,155	700	704
2	20240522	yes	34	300	Yes, 7 px in x direction	1,720	5,024	700	420
3	20240621	yes	34	300	Yes, 7 px in x direction	5,160	2,471	700	88

Table S12. Percentages of gated liposomes (extended data for Fig. S6). Gate > 988 a.u (PAmCherry2 signal). Percentages based on dataset with most events.

Exp	Mix - not activated	Mix - activated
1	0.00%	0.41%
2		0.38%
3	0.00%	0.01%

Table S13. List of primers. Phosphorothioate bonds are indicated with an asterisk (*).

Oligo name	Purpose	DNA sequence (5′ – 3′)
709 ChD	Gene amplification from T7 promoter to T7 terminator; also used for Sanger sequencing of *minD* variants from G598 containing mutations in the MTS of MinD	CAAAAAACCCCTCAAGACCCGTTTAGAG G
757 ChD	Gene amplification from T7 promoter to T7 terminator	TAATACGACTCACTATAGGG
1429 ChD	*PAmCherry2* gene amplification for the assembly of G595	AGCGGCCTGGTGCCGCGCGGCAGCCAT ATGGTGTCTAAAGGGGAAGAGGACAAT
1430 ChD	*PAmCherry2* gene amplification for the assembly of G595	TTTCGGGCTTTGTTAGCAGCCGGATCCTT ACTTATAAAGTTCGTCCATACCCCCCGTT G
1431 ChD	pET15b vector amplification for the assembly of G595	TAAGGATCCGGCTGCTAACAAAGCC
1432 ChD	pET15b vector amplification for the assembly of G595	CATATGGCTGCCGCGCGGCAC
1355 ChD	*mEos2* gene amplification from G571 for the assembly of G577	AGCAGCGGCCTGGTGCCGCGCGGCAGC CATATGAGTGCGATTAAGCCAGACATG
1356 ChD	*mEos2* gene amplification from G571 for the assembly of G577	TCCTTTCGGGCTTTGTTAGCAGCCGGATC CTTATCGTCTGGCATTGTCAGGCAATC
1353 ChD	pET15b vector amplification for the assembly of G577	GGATCCGGCTGCTAACAAAGCCCGAAA G
1354 ChD	pET15b vector amplification for the assembly of G577	ATGGCTGCCGCGCGGCACCAGGCCGCT G
1121 ChD	DNA quantification (qPCR) *YFP* gene	TGCAACTGGCTGACCACTAC
1122 ChD	DNA quantification (qPCR) *YFP* gene	AATGATTGTCCGGCAGCAGA
1208 ChD	DNA quantification (qPCR) *minD* gene	CGCGACTCTGACCGTATTT
1209 ChD	DNA quantification (qPCR) *minD* gene	AGCATGTCACCTCTGCTTAC
1414 ChD	DNA quantification (qPCR) *btubB* gene	CTGACCCTGCAACGTATTCT
1415 ChD	DNA quantification (qPCR) *btubB* gene	TACGGTTCAGTTTCGCCTTC
1445 ChD	Full-plasmid amplification of G437 to introduce mutations in the MTS of MinD	GCCTTTCTTCTCTTCTTCAATGAAGCGGA A
1448 ChD	Full-plasmid amplification of G437 to introduce mutations in the MTS of MinD	T*T*CCGCTTCATTGAAGAAGAGAAGAAA GGCKWMSWCAAACGCTTGTTCGGAGG ATAAGGATCCGGCTGCTAACAAAGCCC
1525 ChD	Forward PCR primer for amplification of *minD* gene	AAAGTAAGCCCCCACCCTCACATGATAC C
1526 ChD	Reverse PCR primer for amplification of *minD* gene	AAAGTAGGGTACAGCGACAACATACACC ATTTCC

Table S14. List of plasmids.

Plasmid name	Description
G365	pUC19-*YFP*
G376	pUC57-*minE_opt*
G437	pUC19-*minD*
G439	pUC19-*btubB*
G595	pET15b-PAmCherry2
G571	pRSET-mEos2
G577	pET15b-mEos2
G598	pUC19-*minD* 32-variant MTS library

DNA sequences

DNA sequence of PAmCherry2

ATGGTGTCTAAAGGGGAAGAGGACAATCTTGCAATCATCAAGGAGTTCATGCGCTTTAAGGTTCATCTGGAA
GGCTCTGTTAATGGACATGAATTTGAAATCGAAGGAGAAGGGGAGGGTCGTCCTTATGAGGGAACTCAAACT
GCTAAATTGAAGGTAACAAAGGGAGGACCACTTCCGTTCGCGTGGGATATTCTGTCGCCCCAGTTCATGTAC
GGAAGCAATGCGTACGTCAAGCATCCCGCTGACATTCCTGACTACTTTAAGCTTTCCTTCCCCGAAGGTTTCA
AATGGGAACGTGTTATGAACTTCGAAGACGGCGGGGTGGTAACTGTGACACAAGATTCGTCGTTGCAAGAC
GGTGAGTTTATCTACAAGGTAAAATTGCGCGGGACAAACTTTCCCTCTGACGGGCCCGTCATGCAAAAAAAG
ACCATGGGGTGGGAAACCTTATCAGAGCGTATGTACCCCGAGGATGGTGCGCTTAAAGGCGAGTTGAAAGC
TCGTACTAAGTTAAAGGATGGCGGACACTATGATACCGAAGTTAAAACGACTTATAAAGCGAAGAAACCCGT
GCAATTACCAGGAGCATACAATGTTAATCGTAAGTTAGACATTACTAGTCACAACGAGGATTATACAATTGTC
GAGCAATACGAGCGTGCTGAGGGTCTTCATTCAACGGGGGGTATGGACGAACTTTATAAGTAA

Degenerate DNA sequence of 32-variant *minD* library with mutations in the MTS

ATGGCACGCATTATTGTTGTTACTTCGGGCAAAGGGGGTGTTGGTAAGACAACCTCCAGCGCGGCCATCGCC
ACTGGTTTGGCCCAGAAGGGAAAGAAAACTGTCGTGATAGATTTTGATATCGGCCTGCGTAATCTCGACCTG
ATTATGGGTTGTGAACGCCGGGTCGTTTACGATTTCGTCAACGTCATTCAGGGCGATGCAACGCTAAATCAG
GCGTTAATTAAAGATAAGCGTACTGAAAATCTCTATATTCTGCCGGCATCGCAAACACGCGATAAAGATGCC
CTCACCCGTGAAGGGGTCGCCAAAGTTCTTGATGATCTGAAAGCGATGGATTTTGAATTTATCGTTTGTGACT
CCCCGGCAGGGATTGAAACCGGTGCGTTAATGGCACTCTATTTTGCAGACGAAGCCATTATTACCACCAACC
CGGAAGTCTCCTCAGTACGCGACTCTGACCGTATTTTAGGCATTCTGGCGTCGAAATCACGCCGCGCAGAAA
ATGGCGAAGAGCCTATTAAAGAGCACCTGCTGTTAACGCGCTATAACCCAGGCCGCGTAAGCAGAGGTGAC
ATGCTGAGCATGGAAGATGTGCTGGAGATCCTGCGCATCAAACTCGTCGGCGTGATCCCAGAGGATCAATCA
GTATTGCGCGCCTCTAACCAGGGTGAACCGGTCATTCTCGACATTAACGCCGATGCGGGTAAAGCCTACGCA
GATACCGTAGAACGTCTGTTGGGAGAAGAACGTCCTTTCCGCTTCATTGAAGAAGAGAAGAAAGGC**KWMS**
WCAAACGCTTGTTCGGAGGATAA

DNA sequence of *minE_opt*

TAATACGACTCACTATAGGGGAATTGTGAGCGGATAACAATTCCCCTCTAGAAATAATTTTGTTTAACTTTAAG
AAGGAGATATACATATGGCGCTGCTGGATTTCTTTCTGAGCCGTAAGAAAAACACCGCGAACATCGCGAAAG
AGCGTCTGCAAATCATTGTTGCGGAGCGTCGTCGTAGCGATGCGGAACCGCACTACCTGCCGCAGCTGCGTA
AAGATATCCTGGAAGTGATTTGCAAGTATGTTCAAATTGACCCGGAGATGGTGACCGTTCAGCTGGAACAAA
AGGACGGTGATATCAGCATTCTGGAGCTGAACGTTACCCTGCCGGAAGCGGAGGAACTGAAGTAAGGATCC
GGCTGCTAACAAAGCCCGAAAGGAAGCTGAGTTGGCTGCTGCCACCGCTGAGCAATAACTAGCATAACCCC
TTGGGGCCTCTAAACGGGTCTTGAGGGGTTTTTG

Supplementary Data 1 (Benchmarking overview) is provided as a separate file.
